# Regulating Employment of Children

**Published:** 1920-04-10

**Authors:** 


### Regulating Employment of Children.

Some sensible draft by-laws regarding child labour have been adopted by the Southport Education Committee, and endorsed by the Town Council. They provide that a child under fourteen shall not, among other things, be employed as a lather boy, or in a similar occupation in a barber's shop; in the kitchen of any hotel, cookshop, eating-house, or refreshment-room; as a marker or attendant in any billiard or bagatelle saloon or other place licensed for games; selling programmes, refreshments, or other articles, taking checks or tickets, or shifting scenery in any theatre, kinematograph hall, or other place of public entertainment; in the collection or sorting of rags, old paper, or refuse; as attendant or assistant in any shop, hall, or other place used for public amusement by means of automatic machines, mutoscopes, shooting ranges, games of chance and skill, or similar devices, or in a slaughterhouse.

A child between twelve and fourteen may be employed on school days between 5 and 7 p.m. (provided that a child employed under a licence in a place of public entertainment shall not have any other employment on the same or following days), or on weekdays, when the school is not open, for not more than five hours, not before 7 a.m. or after 7 p.m.; and on Saturdays the child has to be free for not less than five hours continuously during that period.

A girl under sixteen or boy under fifteen will not be allowed to engage in street trading, and a boy under sixteen only between 7 a.m. and 8 p.m. on weekdays, and not at all on a Sunday. A boy under sixteen so engaged must not enter any premises licensed for public entertainment or public-house for the purpose of trading or delivering goods, and he must have a street trader's licence from the education authority.

Boys and girls under sixteen must not trade at a railway station entrance, and no licensed person, while trading, may be assisted by an unlicensed person under sixteen.

Many of these by-laws indicate an understanding of the kinds of work which may be to the detriment of a child's health, and, further, which cultivate a disagreeably precocious frame of mind or provide hectic experiences to the child's moral and physical disadvantage.

